# Correlation of age with the size of subcortical nuclei of the brain and its implication in degenerative disease: A magnetic resonance imaging study

**DOI:** 10.12688/f1000research.139515.1

**Published:** 2023-09-27

**Authors:** Aditij Dhamija, Lydia S. Andrade, Prakashini K., Chandni Gupta

**Affiliations:** 1 Kasturba Medical College, Manipal Academy of Higher Education, Manipal, Karnataka, 576104, India; 2 Department of Anatomy, Kasturba Medical College, Manipal Academy of Higher Education, Manipal, Karnataka, 576104, India; 3 Department of Radiology, Kasturba Medical College, Manipal Academy of Higher Education, Manipal, Karnataka, 576104, India

**Keywords:** Aging, Magnetic resonance imaging, Neurodegenerative diseases, Brain, Neurosurgeons.

## Abstract

**Background:** Aging is a non-modifiable risk factor for neurodegenerative disease. It is well established that the brain undergoes physiological atrophy with age. So, this study was conducted to analyse the correlation between the age of the person and the size of the various subcortical nuclei of the brain and whether these measurements can serve as a useful indicator for physiological atrophy leading to degenerative disease in clinical practice.

**Methods:** A total of 600 MRI scans from healthy individuals were examined and the measurements of subcortical nuclei were taken and subsequently analysed.

**Results:** A statistically significant difference between the genders was observed in the sizes of the axial diameters of caudate nucleus, putamen and globus pallidus. Caudate nucleus transverse diameter showed a moderate negative correlation with age in males. Globus pallidus axial diameter with age showed weak positive correlation for males. Globus pallidus transverse diameter showed weak positive correlation with age for both males and females, but it was stronger for males compared to females.

**Conclusions:** These results will help neurologists and neurosurgeons in analysing various early degenerative diseases and treat them accordingly.

## Introduction

Aging is a non-modifiable risk factor for neurodegenerative disease. It is well established that the brain undergoes physiological atrophy with age. No single study has yet been conducted on normal people comparing the sizes of all the brain structures together and comparing them with their age.

Decrease in the size of the caudate nucleus is seen in Alzheimer’s disease proportionate to general atrophy of the brain. ^1^ An increase in the size of the caudate nucleus and putamen is noted in patients suffering from Parkinson’s disease. ^2^ It was believed to be due to compensatory changes to account for damage to basal ganglia but a decrease in the size of putamen in both early and advanced Parkinson’s disease and decrease in putamen volume in only advanced Parkinson’s disease is also noted. ^3^ Significantly smaller subcortical nuclei and decreased sizes of caudate nucleus and putamen were seen in people suffering from depression. ^4^ Patients suffering from mild Huntington’s disease showed atrophic changes in the caudate nucleus as well as the putamen, where putamen changes exceed caudate nucleus size changes in early disease. ^5^ Carriers of Huntington’s disease showed smaller sizes of caudate nucleus, putamen and globus pallidus compared to non-gene carriers. ^6^ Patients suffering with late life depression had ventricular enlargement and reduction in the size of the caudate nucleus. ^7^ Basal ganglia sizes (in the caudate nucleus and putamen) are enlarged in patients suffering from schizophrenia. ^8^ Caudate nucleus and putamen may be larger in people suffering from bipolar disorder while they are smaller in those suffering from major depressive disorder. ^9^ Lesch-Nyhan disease was linked with reduction in size of the caudate nucleus and putamen. ^10^ Motor fitness was also linked with an increase in the size of the basal ganglia, and with improved executive function. ^11^

As there are many changes noted in the brain as the ageing process progresses, the size of these structures of the brain will help neurologists and neurosurgeons in analysing various early degenerative diseases and treat them accordingly. This study would be useful in constructing a normogram and the results would help physicians identify certain morphometric changes in size such as those seen in the basal ganglia with schizophrenia patients, which might otherwise be missed.

This study was conducted to analyse the correlation between the age of the person and the size of the various subcortical nuclei of the brain and whether these measurements can serve as a useful indicator of physiological atrophy leading to degenerative disease in clinical practice.

## Methods

### Ethics statement

Institutional ethical clearance was taken before starting the study from Kasturba Medical college and Kasturba Hospital institution ethics committee on 9.2.2021. (IEC 213 – 2021). The only relevant details required were the age and gender of the patient and no other identifying variables were used. This information was collected from the medical records of patients after due permission from the medical superintendent of the hospital and the ethics committee.

We used the STROBE reporting guidelines for our study; a completed checklist is available under Reporting Guidelines. ^12^

### Study design

This was a retrospective study conducted in the Department of Radiology, Kasturba Medical College, Manipal.

### Study size

MRI scans of 600 healthy people were divided into three age groups as follows:

Group 1: 1–18 years

Group 2: 19–40 years

Group 3: 41–70 years

The age of 40 was chosen as studies have indicated that brain volume and weight tends to start decreasing at a rate of 5% from this age. The shrinking of the grey matter is frequently reported after this age. ^13^

### Study period

Two months (1/3/2021–1/5/2021)

### Sample size calculation

The following formula was used to get a combined standard deviation. ^14^

$$\sigma_{c}=\sqrt{\frac{\left(N_{1}-1\right)\sigma_{1}^{2}+\left(N_{2}-1\right)\sigma^{2}+\frac{N_{1}.N_{2}}{N_{1}+N_{2}}\left({\bar{x}}_{1}^{2}+{\bar{x}}_{2}^{2}+2{\overset{=}{x}}_{1}{\bar{x}}_{2}\right)}{N_{1}+N_{2}-1}}$$

The total standard deviation in the 21–60 age range was calculated to be ±1.82mm.

Using the formula for comparison of means ^15^:

$$n=\frac{2\sigma^{2}\left(z_{1-\frac{\alpha }{2}}+z_{1-\beta }\right)}{d^{2}}$$

For 85% power and 99% confidence interval,

$$n=\frac{2{\left(1.82\right)}^{2}\left(2.576+1.037\right)}{{\left(13.29-12.63\right)}^{2}}$$

n=198.53

n≈199

Based on this result, it was decided to keep the sample size of each group as 200 people, for a total sample size of 600 people.

### Inclusion and exclusion criteria

***Inclusion criteria***

(a) Only brain scans belonging to healthy patients were included in the study.
(b) Only brain scans that showed distinct boundaries were considered for measurement.
(c) All patients belonging from 1–70 years were included in the study.

***Exclusion criteria***

(a) All brain scans showing pathological changes affecting the normal anatomy of the basal ganglia were excluded.
(b) All scans showing alteration of the size and shape of the basal ganglia due to any pathological condition were excluded.
(c) All scans with poor positioning of the patients were excluded.
(d) All scans with poor image quality were excluded.
(e) All patients below 1 year and after 70 years were excluded from the study.

## Protocol

***Tools used***

MRI scans were used.

MRI scans were preferred for the following reason:

1. Clear delineation and demarcation of the various structures especially the subcortical nuclei.
2. Ability to visualise structures in different axes allowing for more accurate measurement.
3. Sample is less likely to be misdiagnosed as normal after an MRI scan.

Detailed description of procedure/processes:

The process involved accessing the MRI scans of the patients. The only relevant details required was the age and gender of the patient and no other identifying variable was used. The information was collected from the medical records of patients after due permission from the medical superintendent of the hospital and permission from the ethics committee.

**Figure 1.**
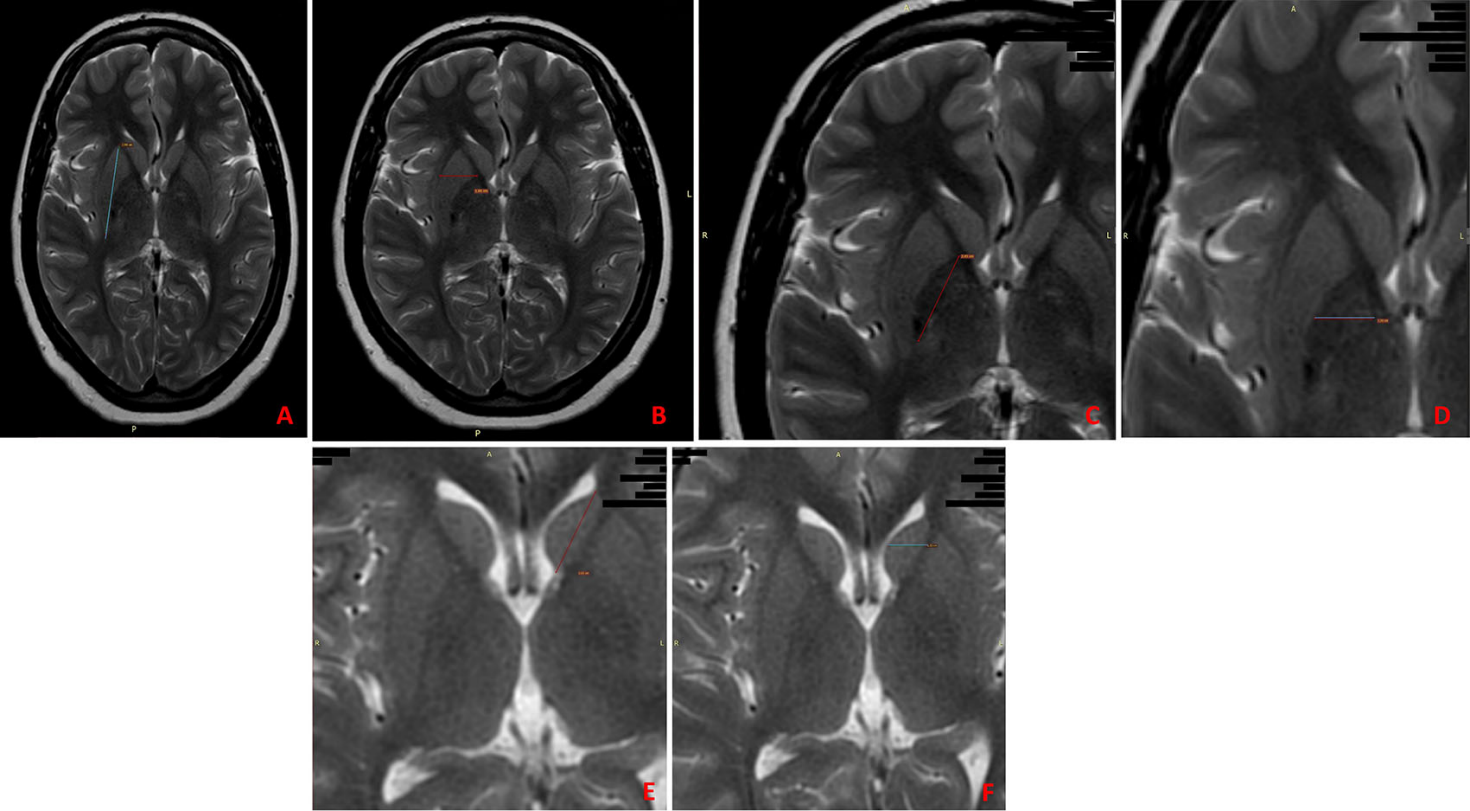
The measurements taken on the subcortical nuclei. A) Vertical/axial diameter of the putamen; B) Transverse diameter of the putamen; C) Vertical/axial diameter of the globus pallidus; D) Transverse diameter of the globus pallidus; E) Vertical/axial diameter of the caudate nucleus; F) Transverse diameter of the caudate nucleus.

The procedure involved the following measurements: ( Figure 1)

1. Measurement of the size of the putamen.
  a) Vertical/axial diameter – Greatest vertical/axial length in mm from upper margin of the putamen to its lower limit.
  b) Transverse diameter – Greatest transverse length in mm along the coronal plane.
2. Measurement of the size of the globus pallidus.
  a) Vertical/axial diameter – Greatest vertical/axial length in mm from upper margin of the globus pallidus to its lower limit.
  b) Transverse diameter – Greatest transverse length in mm along the coronal plane.
3. Measurement of the size of the caudate nucleus.
  a) Vertical/axial diameter - Greatest vertical/axial length in mm from upper margin of the caudate nucleus to its lower limit.
  b) Transverse diameter - Greatest transverse length in mm along the coronal plane.

### Statistical methods

For statistical analysis, IBM SPSS Statistics for Windows Version 20.0 (USA) was used. The mean and standard deviations were calculated for the total population. Spearman’s coefficient was used to evaluate the correlation between age and the size of the parameters under consideration. Spearman’s coefficient was also calculated separately for males and females to judge whether the correlation was more significant for a particular gender with age. We used an ANOVA test to establish whether a significant mean difference exists in the sizes of the parameters for the age groups under consideration. Finally, to establish whether there was any difference in the parameters with regards to sex, we used an independent samples t-test.

Underlying data and statistical analysis is included in the Underlying Data section. ^16^

## Results

The mean and standard deviations of all parameters of the subcortical nuclei are shown in Table 1.

**Table 1.** Mean and standard deviations of all parameters of the subcortical nuclei.

Parameters	Mean	Std. deviation
Caudate nucleus axial diameter	2.0004	0.15142
Caudate nucleus transverse diameter	1.0393	0.12513
Putamen axial diameter	3.9475	0.26488
Putamen transverse diameter	1.39906	0.183268
Globus pallidus axial diameter	2.5384	0.26946
Globus pallidus transverse diameter	1.0001	0.17196

Spearman’s rho for correlation of all the parameters of the subcortical nuclei are shown in Table 2. Spearman’s rho analysis shows a moderate negative correlation between caudate nucleus transverse diameter and age. A weak negative correlation with age was seen with caudate nucleus axial diameter and a weak positive correlation with age was seen with globus pallidus axial diameter and globus pallidus transverse diameter. There was no statistically significant correlation between putamen axial diameters and putamen transverse diameter.

**Table 2.** Spearman’s rho for correlation of all the parameters of the subcortical nuclei.

Parameters		Patient's age
Caudate nucleus axial diameter	Correlation Coefficient	-0.206 ^**^
	Sig. (2-tailed)	0.000
Caudate nucleus transverse diameter	Correlation Coefficient	-0.472 ^**^
	Sig. (2-tailed)	0.000
Putamen axial diameter	Correlation Coefficient	-0.001
	Sig. (2-tailed)	0.984
Putamen transverse diameter	Correlation Coefficient	0.036
	Sig. (2-tailed)	0.376
Globus pallidus axial diameter	Correlation Coefficient	0.222 ^**^
	Sig. (2-tailed)	0.000
Globus pallidus transverse diameter	Correlation Coefficient	0.321 ^**^
	Sig. (2-tailed)	0.000

**Figure 2.**
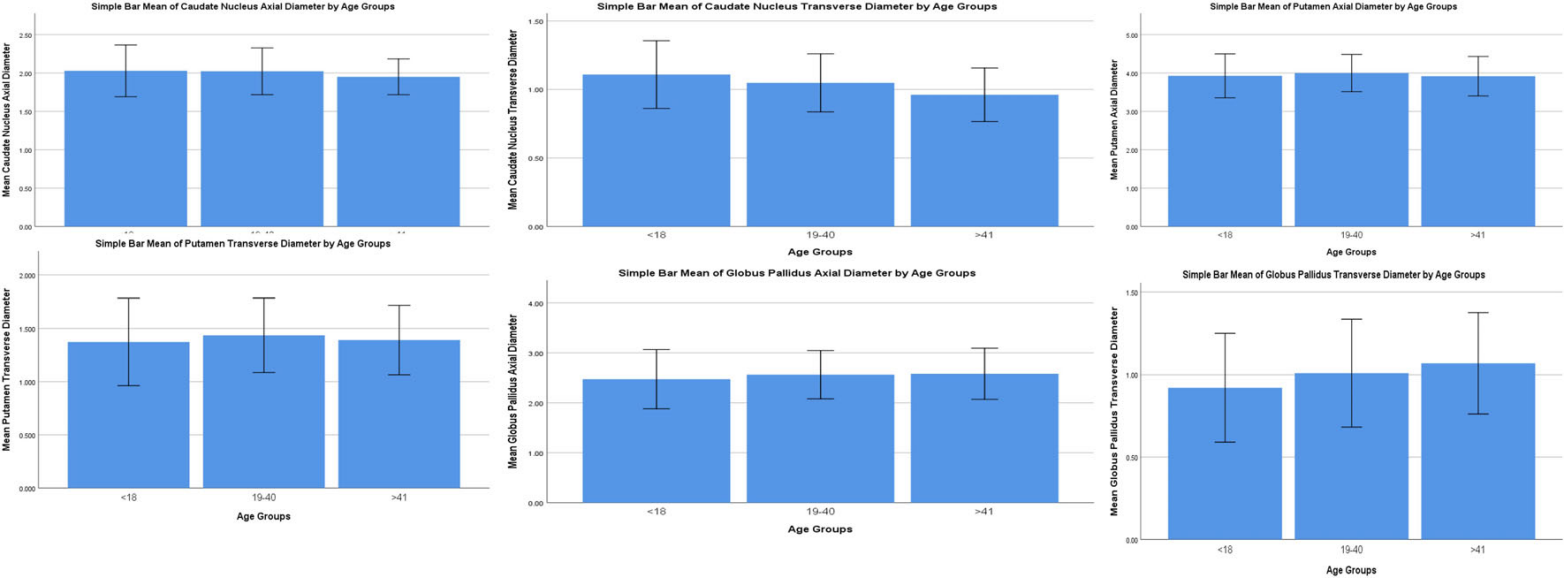
Bar chart visualising the variability of data in the parameters of the subcortical nuclei. The trends seen with age can also be visualised.

Bar means have been constructed to visualise the data with error bars (±2SD) to visualise the variability of data. The trends seen with age can also be visualised. ( Figure 2). Agewise mean and standard deviations of all the parameters of the subcortical nuclei are shown in Table 3. Spearman’s rho for correlation of all the parameters (males and females) are shown in Table 4. In comparing the Spearman’s correlation coefficient between males and females, it was observed that caudate nucleus size showed a greater negative correlation with age in males compared to females. Caudate nucleus transverse diameter showed a moderate negative correlation with age in males and a weaker moderate negative correlation with age in females. Putamen sizes showed an increase with age in males but a decrease with age in females, but these were not found to be statistically significant. Globus pallidus axial diameter with age showed a weak positive correlation for males but it was statistically insignificant for females. Globus pallidus transverse diameter showed a weak positive correlation with age for both males and females, but it was stronger for males compared to females.

**Table 3.** The age wise mean and standard deviations of all the parameters of the subcortical nuclei.

Age groups	<18		19–40		>41		Total	
	Mean	Std. Deviation	Mean	Std. Deviation	Mean	Std. Deviation	Mean	Std. Deviation
Caudate nucleus axial diameter	2.0284	0.16865	2.0224	0.15230	1.9505	0.11662	2.0004	0.15142
Caudate nucleus transverse diameter	1.1087	0.12356	1.0482	0.10600	0.9611	0.09775	1.0393	0.12513
Putamen axial diameter	3.9267	0.28599	3.9993	0.24273	3.9166	0.25765	3.9475	0.26488
Putamen transverse diameter	1.37205	0.205116	1.43450	0.174407	1.39064	0.162917	1.39906	0.183268
Globus pallidus axial diameter	2.4722	0.29650	2.5624	0.24164	2.5808	0.25588	2.5384	0.26946
Globus pallidus transverse diameter	0.9209	0.16513	1.0099	0.16374	1.0696	0.15390	1.0001	0.17196

**Table 4.** Spearman’s rho for correlation of all the parameters (males and females).

Parameters		Patient's age	
		Males	Females
Caudate nucleus axial diameter	Correlation Coefficient	-0.211 ^**^	-0.169 ^**^
	Sig. (2-tailed)	0.000	0.005
Caudate nucleus transverse diameter	Correlation Coefficient	-0.493 ^**^	-0.436 ^**^
	Sig. (2-tailed)	0.000	0.000
Putamen axial diameter	Correlation Coefficient	0.141 ^*^	-0.143 ^*^
	Sig. (2-tailed)	0.011	0.017
Putamen transverse diameter	Correlation Coefficient	0.137 ^*^	-0.099
	Sig. (2-tailed)	0.013	0.102
Globus pallidus axial diameter	Correlation Coefficient	0.295 ^**^	0.149 ^*^
	Sig. (2-tailed)	0.000	0.013
Globus pallidus transverse diameter	Correlation Coefficient	0.361 ^**^	0.281 ^**^
	Sig. (2-tailed)	0.000	0.000

**Figure 3.**
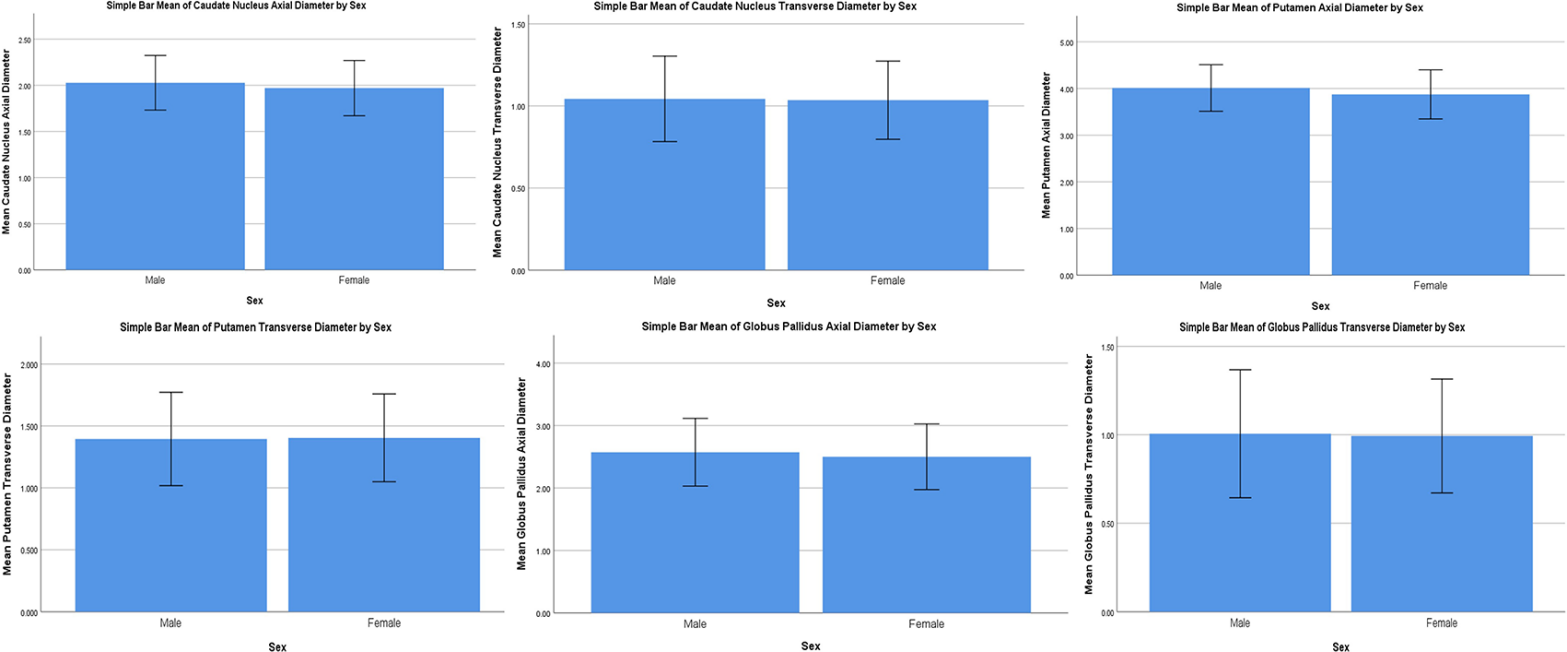
Bar chart visualising the variability of data in the parameters of the subcortical nuclei. The trends between males and females can be observed for all parameters.

Mean bars have been constructed to visualise the data with an error bar (±2SD) to visualise the variability of data. The trends between males and females can be observed for all parameters. ( Figure 3). Analysis of variance of all parameters between groups is shown in Table 5. All parameters showed significance. This shows that a difference in size exists between the three age groups for the axial and transverse diameters of the caudate nucleus, axial and transverse diameters of the putamen, axial and transverse diameters of the globus pallidus.

**Table 5.** Analysis of variance of all parameters between groups in total males and females.

	Total		Males		Females	
Parameters	F	Sig.	F	Sig.	F	Sig.
Caudate nucleus axial diameter	17.282	0.000	15.371	0.000	4.166	0.017
Caudate nucleus transverse diameter	91.672	0.000	57.828	0.000	33.686	0.000
Putamen axial diameter	5.897	0.003	8.438	0.000	5.276	0.006
Putamen transverse diameter	6.230	0.002	3.647	0.027	6.930	0.001
Globus pallidus axial diameter	9.561	0.000	11.912	0.000	1.318	0.269
Globus pallidus transverse diameter	43.174	0.000	26.869	0.000	16.869	0.000

Analysis of variance for all parameters for males is shown in Table 5. All parameters showed significance for males. This shows that a difference in size exists between the three age groups for males for the axial and transverse diameters of the caudate nucleus, axial and transverse diameters of the putamen, axial and transverse diameters of the globus pallidus *.*

Analysis of variance for all parameters for females is shown in Table 5. All except globus pallidus axial diameter is significant for females. This shows that a difference in size exists between the three age groups for females for the axial and transverse diameters of the caudate nucleus, axial and transverse diameters of the putamen, transverse diameter of the globus pallidus.

The final results show that caudate nucleus axial diameter showed a general decrease in size with age, but in men it showed a slight increase in size when the <18 age group was compared to the 19–40 age group which was not found to be statistically significant. This increase was offset by the greater decrease in size from the 19–40 age group and therefore the decrease in the size of caudate nucleus axial diameter was found to be greater in males compared to females. The difference of means is significant for the <18 and >41 age group and 19–40 and >41 age groups in males and for the <18 and >41 age group in females.

The decrease in size of the caudate nucleus transverse diameter seems to be greater in males compared to females. The difference of means is significant for all three age groups in both genders.

Putamen axial diameter sizes seems to increase in males from <18 to 19–40 age group and then shows a slight decrease from 19–40 to >41 age group but the decrease is not significant enough to offset the initial increase. In females, it showed a minor increase from <18 to 19–40 which was not statistically significant and then decreased from 19–40 to >41 which was significant enough to offset the initial increase. This difference of means is significant in the <18 and 19–40 age group in males and for <18 and >41 age group and 19–40 and >41 age group in females.

Putamen transverse diameter sizes seem to increase in males in the <18 and 19–40 age group and shows a very slight decrease from the 19–40 to >41 age group, none of which are found to be statistically significant. In females, an initial increase in size is seen from the 19–40 to >41 age group followed by a significant decline from the 19–40 to >41 age group which is enough to offset the initial increase. The difference of means is significant in the 19–40 and >41 age group in females.

Globus pallidus axial diameter seems to rise significantly in males. There is a marked increase in size from the <18 age group and the 19–40 age group, while the increase in size in the 19–40 to >41 age group is insignificant. In females, the increase in size is comparatively insignificant. The difference of means is significant only for males in the <18 and 19–40 and the <18 and >41 age groups.

Globus pallidus transverse diameter seems to rise in both males and females with the rise being slightly higher in men. The difference of means is significant for males in all three age groups and for females in the <18 and 19–40 age group and the <18 and >41 age groups.

Group statistics for all parameters (genderwise) is shown in Table 6. An independent t-test was done to compare all parameters between genders. A statistically significant difference between the genders was observed in the sizes of the caudate nucleus axial diameter (P value: 0.000), putamen axial diameter (P value: 0.000), globus pallidus axial diameter (P value: 0.001). However, no statistically significant differences in sizes of the structures with genders was found between the caudate nucleus transverse diameter, putamen transverse diameter and globus pallidus transverse diameter.

**Table 6.** Group statistics for all parameters (Genderwise).

Parameters	Sex	N	Mean	Std. Deviation	Std. Error Mean
Caudate nucleus axial diameter	M	324	2.0269	0.14803	0.00822
	F	276	1.9692	0.14965	0.00901
Caudate nucleus transverse diameter	M	324	1.0430	0.13018	0.00723
	F	276	1.0350	0.11902	0.00716
Putamen axial diameter	M	324	4.0107	0.24962	0.01387
	F	276	3.8733	0.26345	0.01586
Putamen transverse diameter	M	324	1.39493	0.188420	0.010468
	F	276	1.40391	0.177247	0.010669
Globus pallidus axial diameter	M	324	2.5717	0.27093	0.01505
	F	276	2.4993	0.26288	0.01582
Globus pallidus transverse diameter	M	324	1.0058	0.18087	0.01005
	F	276	0.9934	0.16095	0.00969
	F	276	2.8670	0.27474	0.01654

## Discussion

Our study found a general decrease in the size of the caudate nucleus. The decrease in the size of the caudate nuclei with aging supports the findings of Amal *et al*. and Gunning-Dixon *et al*. ^17^^,^^18^ The correlation with age was strongest for caudate nucleus transverse diameter, which showed a decrease with aging. The caudate nucleus axial diameter showed weaker correlation with a general decrease in size with age, but in men, it showed a slight increase in size from the <18 age group to the 19–40 age group, which was not found to be statistically significant. This increase was offset by the greater decrease in size from the 19–40 age group and therefore the decrease in the size of caudate nucleus axial diameter was found to be greater in males compared to females. The difference of means is significant for the <18 and >41 age group and 19–40 and >41 age groups in males and for the <18 and >41 age group in females. The apparent increase in size in men may be due to similar sizes being seen in the <18 and 19–40 age group, which may result in an apparent increase in length due to inherent physiological variations in the two samples. Hence, the increase is not found to be statistically significant. The decrease in the size of caudate nucleus transverse diameter also seems to be greater in males compared to females. The difference of means is significant for all three age groups in both genders. The caudate nucleus transverse diameter showed no significant differences between the genders. This supports the findings of Rijpkema *et al*., who found no significant difference between the genders. ^19^ However, a significant difference was found in the axial diameter of the caudate nucleus. Due to the transverse diameter being non-significant for gender, the volumetric relationship might become non-significant for gender and thus explain the results of Rijpkema *et al*. ^19^

For both putamen axial and transverse diameter, a decrease in size is seen in both genders in the 19–40 and >41 age group. This supports the findings reported by Amal *et al*., Gunning-Dixon *et al*. and Walhovd *et al*. with regards to a decrease in the size of the putamen with age in the elderly. ^17^^,^^18^^,^^20^ In our study, putamen sizes showed an initial increase in size in both genders from the <18 to 19–40 age group which was statistically significant for men. The initial increase in size in men was not compensated for by the subsequent decrease in size and hence overall the size of the putamen was found to have increased. The causes for this finding and what implications it may have can serve as grounds for future research on this topic. Our study is also able to confirm the finding of an increased size of the putamen in men as was reported by Rijpkema *et al*. ^19^

Globus pallidus axial diameter seems to rise significantly in males. There is a marked increase in size from the <18 age group and the 19–40 age group, while the increase in size in 19–40 to >41 age group is insignificant. In females, the increase in size is comparatively insignificant. The difference of means is significant only for males in the <18 and 19–40 and the <18 and >41 age group. Globus pallidus transverse diameter seems to rise in both males and females with the rise being slightly higher in men. The difference of means is significant for males in all three age groups and for females in the <18 and 19–40 age group and the <18 and >41 age group. This is different from the result found by Gunning-Dixon *et al*. and Brabec *et al*. ^18^^,^^21^ This may be due to differences in the method and site of measurements as well as the boundaries of the structure considered. Globus pallidus axial diameter was shown to have significant differences between the genders, which supports the finding of Rijpkema *et al*. who found a significant larger size of the globus pallidus in males. ^19^ The transverse diameter showed no significant difference between the genders.

The data gathered from this research will be a useful tool for the clinician to assess radiological scans and detect neurodegenerative disorders early in a patient.

## Conclusions

In this study, we examined the correlation between age and the sizes of the basal ganglia. A total of 600 MRI scans from healthy individuals were examined and the measurements were taken and subsequently analysed.

In males as the age advances caudate nucleus transverse diameter decreases, globus pallidus axial diameter increases. In both males and females globus pallidus transverse diameter increases with age. All dimensions of caudate nucleus, putamen and globus pallidus were found to be larger in males.

AS the age advances the putamen transverse length showed an increase in size in males and a decrease in size in females, which could have clinical ramifications that should be explored in more depth in future studies.

One of the limitations of the study is that it utilised scans from a single hospital in South India, therefore, similar studies should be performed in other centres so that the findings can be generalised for the entire population.

## Data Availability

Figshare: Underlying data for ‘Correlation of age with the size of subcortical nuclei of the brain and its implication in degenerative disease: A magnetic resonance imaging study’. https://www.doi.org/10.6084/m9.figshare.23634468. ^12^ Figshare: STROBE checklist for ‘Correlation of age with the size of subcortical nuclei of the brain and its implication in degenerative disease: A magnetic resonance imaging study’. https://www.doi.org/10.6084/m9.figshare.23641866. ^16^ Data are available under the terms of the Creative Commons Attribution 4.0 International license (CC-BY 4.0)
