# Supplementary material for: Correlation of age with the size of subcortical nuclei of the brain and its implication in degenerative disease: A magnetic resonance imaging study
Source: F1000Res. 2024 Apr 2;12:1230. Originally published 2023 Sep 27. [Version 2] doi: 10.12688/f1000research.139515.2 (PMC11061590; doi:10.12688/f1000research.139515.2)
Supplement: Peer review history

# Reviewer report: Reviewer response for version 2

**DOI:** 10.5256/f1000research.164295.r264570

**Published:** 2024-04-30

**Authors:** Jolly Agarwal, Anurag Agrawal

**Affiliations:** 1 Pulmonary Medicine, Government Doon Medical College, Dehradun, Uttarakhand, India; 2 Government doon medical college, Dehradun, India

**Strength of the article-**

 The present article focuses on statistically significant gender wise differences in the subcortical nuclei of the brain by MRI.Till date there is less research in this area.It can help in understanding and analysing various early degenerative diseases of the brain. It can be done in more centres across countries or states so that observations can be used to make policy.

**Summary-**

 The present study focuses on correlation of age with size of subcortical nuclei.It concludes that there is gender wise as well as age wise difference in ageing of sub cortical nuclei.This meticulous study can help neuro physician and neurosurgeon to do better.The present study find out that there is statistically significant difference in axial diameters of caudate nucleus, putamen and globus pallidus in male and female. It also emphasises correlation of sub cortical nuclei diameters with age.

**Major points-**

 In the present study, the authors observed MRI images of 600 healthy individuals. The age of individuals were from 1-70 years and they were divided into three age groups. A statistically significant difference was observed in the size of the caudate nucleus axial diameter, putamen axial diameter, globus pallidus axial diameter between the genders,While statistically significant differences in sizes of the structures with gender was not found between the caudate nucleus transverse diameter, putamen transverse diameter. The decrease in size of the caudate nucleus transverse diameter seems to be greater in males compared to females. The difference of means is significant for all three age groups in both genders.

**Minor points-**

 A weak negative correlation with age was seen with caudate nucleus axial diameter and a weak positive correlation with age was seen with globus pallidus axial diameter and globus pallidus transverse diameter. Caudate nucleus size showed a greater negative correlation with age in males compared to females.Globus pallidus transverse diameter showed a weak positive correlation with age for both males and females, but it was stronger for males compared to females.

Is the work clearly and accurately presented and does it cite the current literature?

Yes

If applicable, is the statistical analysis and its interpretation appropriate?

Yes

Are all the source data underlying the results available to ensure full reproducibility?

Yes

Is the study design appropriate and is the work technically sound?

Yes

Are the conclusions drawn adequately supported by the results?

No

Are sufficient details of methods and analysis provided to allow replication by others?

Yes

Reviewer Expertise:

Radiology, Histology, Medical education, Gross Anatomy, Osteology

We confirm that we have read this submission and believe that we have an appropriate level of expertise to confirm that it is of an acceptable scientific standard.

# Reviewer report: Reviewer response for version 2

**DOI:** 10.5256/f1000research.164295.r261805

**Published:** 2024-04-04

**Authors:** Vidya CS JSSMC00341

**Affiliations:** 1 JSS Academy of Higher Education and Research, Karnataka, India

All the reviewers comments has been addressed

 The article is approved for further processing

Is the work clearly and accurately presented and does it cite the current literature?

Yes

If applicable, is the statistical analysis and its interpretation appropriate?

Yes

Are all the source data underlying the results available to ensure full reproducibility?

Partly

Is the study design appropriate and is the work technically sound?

Partly

Are the conclusions drawn adequately supported by the results?

Yes

Are sufficient details of methods and analysis provided to allow replication by others?

Yes

Reviewer Expertise:

Potential biomarkers in Alzheimer's disease

I confirm that I have read this submission and believe that I have an appropriate level of expertise to confirm that it is of an acceptable scientific standard.

# Reviewer report: Reviewer response for version 1

**DOI:** 10.5256/f1000research.152795.r254310

**Published:** 2024-03-25

**Authors:** Vidya CS JSSMC00341

**Affiliations:** 1 JSS Academy of Higher Education and Research, Karnataka, India

**Strength of the article:** • Study has used 600 Scans which are a huge sample. • They have measured all parts of the basal ganglia- Caudate nucleus, putamen and globus pallidus. Weakness of the article: • This study utilised scans from a single hospital in South India, therefore, similar studies should be performed in other centres so that the findings can be generalised for the entire population. 

**Summary • **This study proves the correlation with age of the basal ganglia, and finds that gender plays a significant role, and charts the differential aging of the structures in the two sexes. • The data gathered from this research will be a useful tool for the clinician to assess radiological scans and detect neurodegenerative disorders early in a patient. 

**Major points **• In this study, they examined the correlation between age and the sizes of the basal ganglia. A total of 600 MRI scans from healthy individuals were taken. • Globus pallidus axial diameter with age showed weak positive correlation for males. Globus pallidus transverse diameter showed weak positive correlation with age for both males and females but it was stronger for males compared to females. • A statistically significant difference between the genders was observed in the sizes of the axial diameters of caudate nucleus, putamen and globus pallidus. All of these structures were found to be larger in males. 

**Minor points **• The caudate nucleus showed a significant decrease in size, which can be a result of physiological atrophy. • The putamen transverse length showed an increase in size in males and a decrease in size in females.

Is the work clearly and accurately presented and does it cite the current literature?

Yes

If applicable, is the statistical analysis and its interpretation appropriate?

Yes

Are all the source data underlying the results available to ensure full reproducibility?

Partly

Is the study design appropriate and is the work technically sound?

Partly

Are the conclusions drawn adequately supported by the results?

Yes

Are sufficient details of methods and analysis provided to allow replication by others?

Yes

Reviewer Expertise:

Potential biomarkers in Alzheimer's disease

I confirm that I have read this submission and believe that I have an appropriate level of expertise to confirm that it is of an acceptable scientific standard, however I have significant reservations, as outlined above.

# Response

**Published:** 2024-03-26

**Authors:** Chandni Gupta

**Affiliations:** Anatomy, Kasturba Medical college, Manipal, Manipal, Karnataka, India

Dear Madam,

 In materials and Methods: I have added the MRI machine which was used to take scans of patients.

 And in analysis part I have added that measurements were taken with the help of radiology experts and even the measurements were taken twice to prevent interobserver variability.

 Rest all remains same.
